# Remarkable Shifts in Offspring Provisioning during Gestation in a Live-Bearing Cnidarian

**DOI:** 10.1371/journal.pone.0154051

**Published:** 2016-04-22

**Authors:** Annie Mercier, Zhao Sun, Christopher C. Parrish, Jean-François Hamel

**Affiliations:** 1 Department of Ocean Sciences, Memorial University, St. John’s, Newfoundland and Labrador, Canada; 2 Society for the Exploration and Valuing of the Environment (SEVE), Portugal Cove-St. Philips, Newfoundland and Labrador, Canada; Universidad de Granada, SPAIN

## Abstract

Animals display diverse means of producing and provisioning offspring, from eggs to embryos and juveniles. While external development predominates, many forms of embryonic incubation have evolved, including placentation in mammals and a number of understudied variants in basal metazoans that could help understand evolutionary diversification. Here we studied the brooding sea anemone *Aulactinia stella*, using behavioural, morphological and biochemical indicators of offspring phenotype to characterize gestation and elucidate parental and sibling relationships. The pronounced variance in juvenile weight within broods was not strongly related to any of the typical external predictors (adult weight, clutch size, sampling date, environmental conditions). Lipid concentration was significantly higher in the tissues of the small juveniles than in those of large juveniles or adult, and fatty acid profiles tended to set small juveniles apart. Finally, intra-brood feeding on external resources was documented in larger juveniles. These results are consistent with ontogenetic shifts in nutrition, from vitellogenic provisioning to post-zygotic nourishment to a prenatal form of nursing upon acquisition of feeding organs, highlighting matrotrophic and conflict-driven mechanisms acting on offspring phenotype during gestation.

## Introduction

The many different ways through which animals produce and nurture their progeny have long been a source of curiosity and wonder. A continuum of strategies, from external development involving minimal parental care all the way to direct nutrient transfer across placental tissues, have been documented [1–5]. Evolutionary transitions towards the retention of progeny are typically interpreted to favour survival of incubated offspring [6]. The effects of parent-offspring conflicts and sibling rivalries are less often explored, even though they are expected to increase with the length or intimacy of parental care [2, 7, 8]. Competition among siblings is most often studied in species with postnatal nursing, such as birds and mammals [9], but it may also be important when parental care occurs between fertilization and birth/release [10–13].

Forms of offspring incubation (live-bearing/viviparity or brooding) involving lecithotrophy (vitellogenic nourishment through yolk reserves) or matrotrophy (extra-vitellogenic nourishment by the parent) are reported in members of most animal phyla, including but not restricted to chordates, echinoderms, molluscs, arthropods, bryozoans and cnidarians [4, 14]. In brooding and viviparous species alike, competition among sibling embryos for space and maternally-derived resources may arise, which will likely have a profound impact on offspring phenotype. Importantly, the advent of matrotrophy marks a crucial shift in the timing of resource allocation to parental care; from pre-fertilization in lecithotrophic species to a more evenly spread investment throughout gestation in matrotrophic species. Thus, studies of offspring incubation in basal metazoans may provide significant insight when developing evolutionary concepts of reproductive strategies, as shown in investigations of mating systems [15].

The present study focused on a species belonging to a basal animal phylum, the sea anemone *Aulactinia stella* (Cnidaria: Actiniaria), which incubates progeny in the gastrovascular cavity (coelenteron) and releases few fully-formed benthic juveniles [16, 17]. The intimacy of the relationship between the mother and its progeny before birth can be difficult to ascertain and define. Offspring size metrics and incubation site (e.g. in reproductive *vs* digestive cavity) may be used to infer matrotrophy and distinguish viviparity from brooding [4], whereas chemical markers may help assess nutrition sources (e.g. yolk *vs* diet). Lipids have commonly been used as trophic markers to provide information on dietary intake [18] and to assess the use of nutritive reserves during offspring development [19–21].

The objectives of this study were to (1) characterize the brooding process in *A*. *stella* by long-term monitoring, (2) assess development level and size of juveniles during incubation and at release, relative to clutch size, parent size, season and environment (field *vs* laboratory), (3) compare lipid and fatty acid profiles in adult tissues and juveniles of different sizes, and (4) use lipid signatures to detect any shift from maternally-derived to dietary nutritional resources during early ontogeny.

## Materials and Methods

Adults of *A*. *stella* were collected at a depth of ~10 m off the Avalon Peninsula, Newfoundland, Canada (47°17’44.6”N, 52°46’8.9”W) from March-July 2009, March-June 2010, and in January 2011. Individuals were distributed in flow-through holding tanks (20 L) for short-term storage before being transferred into experimental units (see below). Each holding tank held 6–10 individuals, and was supplied with unfiltered running seawater (~8 L min^-1^), at ambient temperature (0–10°C), under natural photoperiod and plankton food supply. Dive collections were performed by the Field Services of the Department of Ocean Sciences with the required permits from Fisheries and Oceans Canada.

### Brooded juveniles in freshly collected adults

Forty adults were examined within 3 days of collection in March-June 2010 and January 2011 to estimate reproductive activity and natural size variation of incubated offspring inside brooding adults. Adult wet weight (after removing juveniles and incising the basal disk to drain excess water), basal disk diameter and contracted height were measured. Each specimen was dissected by removing the basal disk and cutting vertically along the mesenteries. The presence of gamete-bearing mesenteries, i.e. oogenic mesenteries, was noted and numbers of juveniles were recorded on removal. Juvenile wet weight and volume (basal area × contracted height) were measured immediately after extraction. In addition, subsamples from four adults were collected and preserved for lipid and fatty acid analysis (see below).

### Comparison of offspring phenotypes during incubation and at release

Adults of *A*. *stella* were reared individually in 2-L flow-through containers for long-term monitoring of the release of juveniles from June 2009-March 2010 (n = 8) and April 2010-April 2011 (n = 8). All containers were supplied with unfiltered running seawater (~1.5 L min^-1^), at ambient temperature under natural photoperiod and plankton food supply. Urchin gonads or shrimp (~0.5 g) were fed into the mouth of the sea anemones every other week. The natural release of juveniles by each brooding adult of *A*. *stella* was monitored weekly and wet weight (an accurate measurement of *A*. *stella* juvenile size; see results) measured as described earlier. At the end of both experimental periods (March 2010 and April 2011), all adults (n = 16) were dissected as described above to assess brooding status. Wet weight of adults as well as number and wet weight of any incubated juveniles were also measured as described above.

### Feeding experiment

During a preliminary study, some *A*. *stella* juveniles were observed with their tentacles extended while being extracted from brooding adults. Thus, feeding experiments were conducted to test whether juveniles were capable of feeding on food obtained by the brooding adult (while nestling inside the gastrovascular cavity or along the mesenteries). Before the experiment, six adults (10.2–56.0 g) were transferred into separate 2-L containers under low flow (~0.5 L min^-1^) and acclimatized overnight. Shrimp was used in the feeding experiment because individuals of *A*. *stella* had shown active feeding on shrimp fragments and the shrimp brightness made it easy to distinguish visually whether juveniles (translucent beige or greenish) were feeding on food ingested by the brooding adult. Shrimp paste (2 ml) was dropped on the tentacles close to mouths of adults hourly for 6 consecutive hours. Adults were left overnight to provide enough time for full ingestion. They were examined 24 h after first feeding, as described above. All juveniles inside the brooding adult were collected and transferred to a Petri dish and the number and weight of positively-feeding juveniles, i.e. those with traces of food in their gastrovascular cavity, was recorded.

### Lipids and fatty acid signatures

To compare lipid composition of adults and offspring, samples were collected of adult body wall (n = 11 from 4 adults, 2~3 samples per adult, from the basal disk) and oogenic mesenteries (n = 9 from 3 adults), and of whole juveniles of various sizes (n = 12 from 4 clutches) in May-June 2010. Oogenic mesenteries were collected from the only three individuals with such tissue. Twelve juveniles were divided into two size classes to compare lipid composition, with small juveniles (n = 6) weighing 7–77 mg and large juveniles (n = 6) 122–308 mg. Samples were preserved in 2 ml chloroform under N_2_ at -20°C for lipid and fatty acid analyses. Fatty acids were determined in the three individuals that possessed gametes. For juvenile samples, only the smallest and largest juvenile from each brood/adult were analysed. The small juvenile class (n = 3) weighed 8–77 mg, and the large juvenile class (n = 3) weighed 186–308 mg.

Extraction and analysis of lipids were based on standard methods [22]. Lipid classes were determined using thin layer chromatography with flame ionization detection with a MARK V Iatroscan (Iatron Laboratories, Tokyo, Japan). Data were processed using the PeakSimple Chromatography software (V3.88, SRI Instruments, US). Fatty acid methyl esters (FAME) were analysed on a HP 6890 GC FID equipped with a HP 7683 autosampler. Peaks were identified using retention times from standards purchased from Supelco: 37 component FAME mix, Bacterial acid methyl ester mix, PUFA 1 and PUFA 3. Chromatograms were integrated using the Varian Galaxie Chromatography Data System, version 1.9.3.2. The Iatroscan determined derivatization efficiency for the samples was 76%. Lipid data are reported as % weight.

### Data analysis

Relationships between juvenile weight and volume, and between different juvenile variables and parent weight or clutch size were determined using Spearman’s rank order correlation. Within-brood variance (CV) of mean weight was compared between life stages (during incubation, at release), between years, and between environments (laboratory, field) using *t*-tests, or nonparametric Mann-Whitney *U* tests where assumptions of equal variance failed. One-way ANOVA (or Kruskal-Wallis test) was used to test the influence of sampling month on mean and variance of juvenile weight.

Weights of juveniles with and without traces of feeding were compared with *t*-tests. Lipid and fatty acid proportions were analysed by ANOVA, or Kruskal-Wallis test. Major fatty acids (> 1%) in adult body wall, oogenic mesentery, and large and small juveniles were compared using the Bray-Curtis similarity measurement and non-metric multi-dimensional scaling (MDS) analyses [23]. Variation in fatty acid composition among types of samples was subsequently tested for significance with ANOSIM (analysis of similarities) [23]. The R_ANOSIM_ statistic values varied from 0 (no difference among groups) to 1 (samples within the same group are more similar than samples from different groups). SIMPER (similarity percentage analysis) [23] was used to explore the relative contribution of individual fatty acids to dissimilarity among different types of samples.

## Results

**Fig 1:**
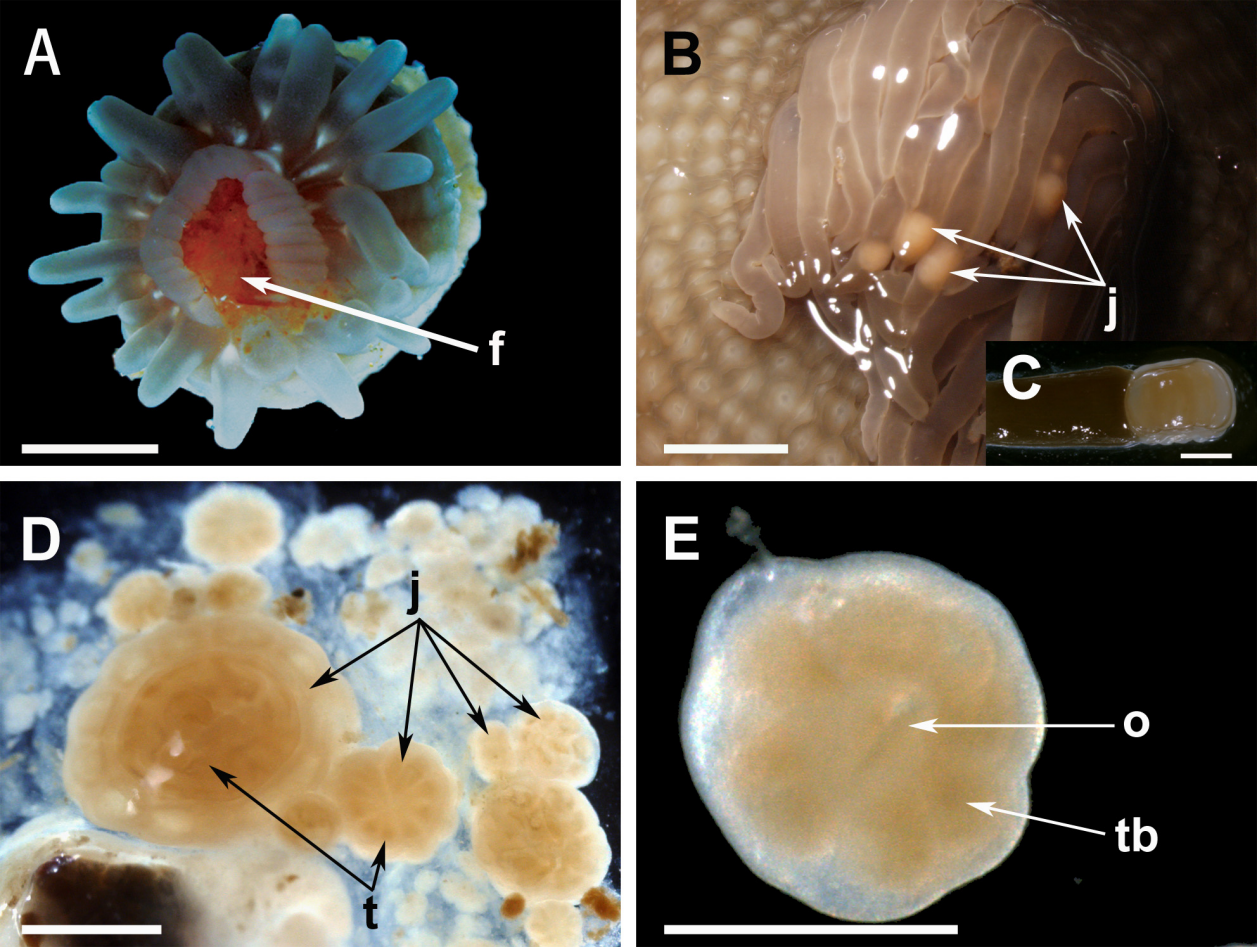
Details of *Aulactinia stella*. (A) Brooded juvenile; this one was scored as positive for intra-brood feeding based on presence of food (f) in the gastrovascular cavity. (B) Small juveniles (j) moving freely in the tentacles of a brooding adult. (C) Close-up of a small juvenile in B. (D) Size variation of offspring released in a mucus bundle, including tiny propagules and metamorphosing juveniles (j), with primary tentacles (t). (E) Close-up of a small metamorphosing juvenile in D, showing oral pore (o) and tentacle buds (tb). Scale bar represents 2 mm in A, 4 mm in B, 1 mm in C and D, and 0.5 mm in E.

Like other sea anemones, *A*. *stella* lacks discrete ovaries, and oocytes grow within reproductive mesenteries between the retractor muscle and mesenterial filaments. While *A*. *stella* is sometimes listed as a protandric hermaphrodite [24], parthenogenetic reproduction is also reported [16]. No spermatozoa were detected in the sea anemones studied here (*N* = 56). Juveniles of *A*. *stella* were brooded freely inside the gastrovascular cavity, and typically emitted individually through the mouth from August to October. Fully developed juveniles (Fig 1A) up to 312 mg were released. Small juveniles (~5 mg) were also observed in the tentacles of three adults (Fig 1B and 1C). Furthermore, two adults were seen to release ~25 tiny propagules (< 5 mg) in mucus bundles through the mouth (Fig 1D) or individually through tentacle tip pores (approximately 60% of these were < 1 mg). Unlike typical juveniles, these propagules, especially those < 1 mg, were covered with cilia, and were able to move rapidly in seawater. They had septa but their mouth and tentacles were not well-developed (Fig 1E).

### Offspring phenotype during parental care in the field

**Fig 2:**
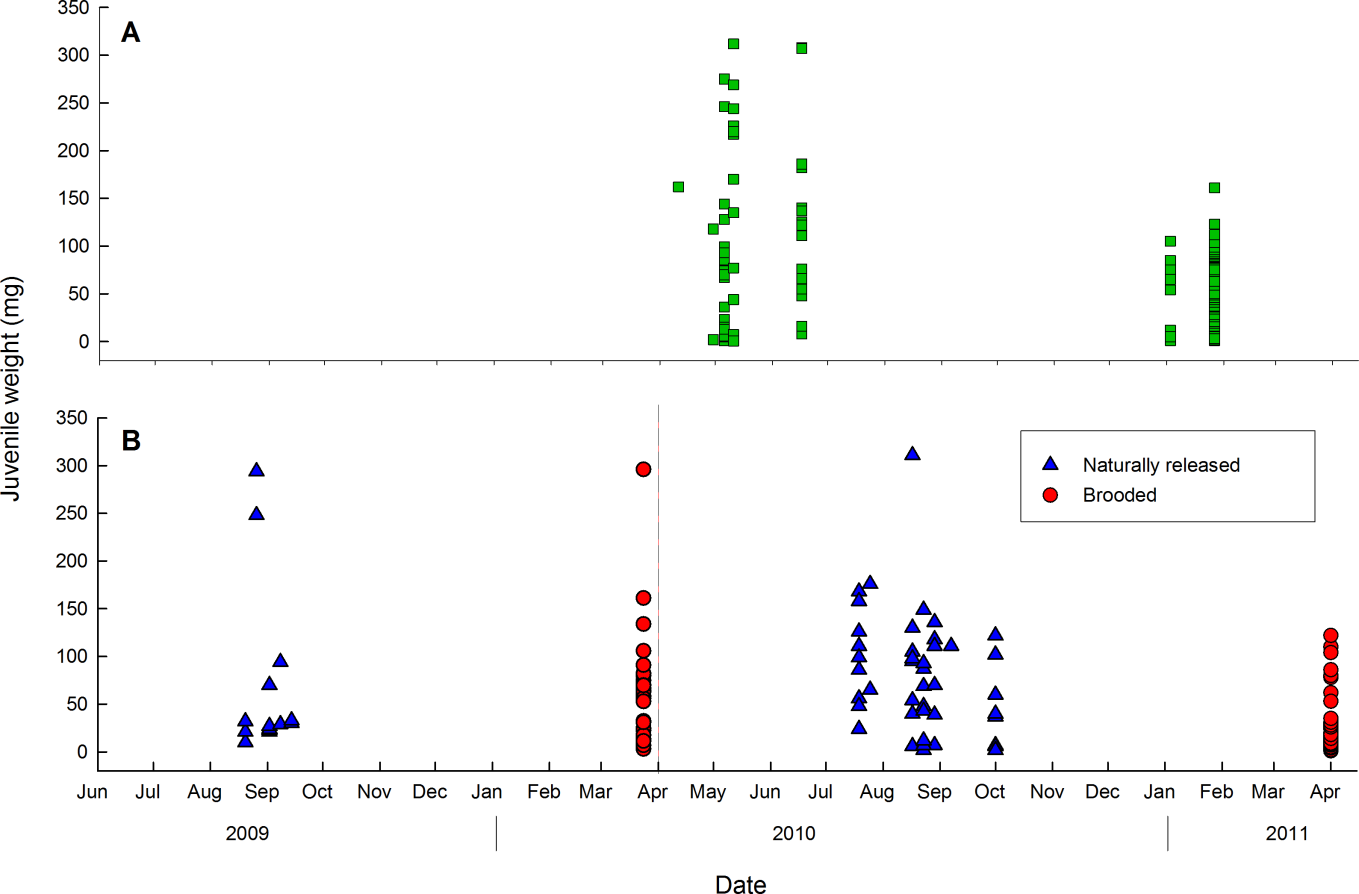
Distribution of juvenile sizes (wet weight) over time in *Aulactinia stella*. (A) Juveniles extracted from the gastrovascular cavity immediately after collection of brooding adult. (B) Naturally-released juveniles and brooded juveniles in two experimental periods from 2009 to 2011. Dashed line separates the two experimental periods.

Among the 40 adults (1.1–56.0 g) examined immediately after collection in April-May-June 2010, and January 2011, a total of 25 (62.5%) were brooding juveniles (Table A in S1 File). The proportion of brooding adults fluctuated from 50.0–88.9% in the four sampling months. Wet weights of 179 juveniles extracted from the brooding adults varied from 0.5 to 312 mg (Fig 2), with a mean of 59.3 mg and their volume varied from 0.4 to 395.2 mm^3^, with a mean of 58.8 mm^3^. The weight of juveniles was significantly correlated with their volume (*r*_*s*_ = 0.94, *N* = 179, *P* < 0.005) and thus was considered an accurate measurement of size.

**Fig 3:**
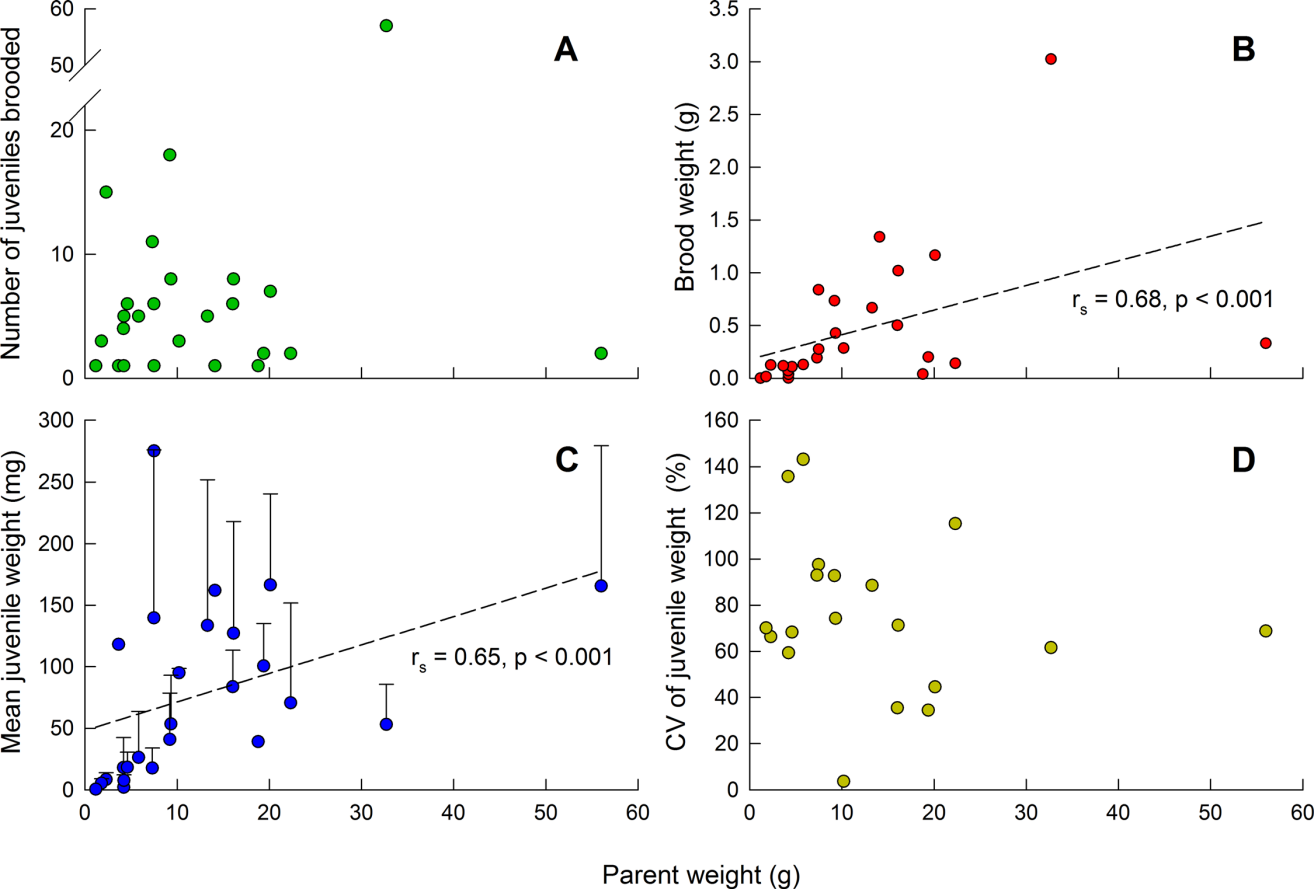
Influence of adult wet weight on various metrics in *Aulactinia stella*. (A) Clutch size (number of juveniles being brooded); (B) wet weight of entire brood (g); (C) mean (+SD) juvenile wet weight (mg); and (D) within-brood CV of mean juvenile weight.

Clutch size (number of juveniles per brood) varied from 1 to 57 (Table A in S1 File), with a majority ≤ 15 and a mean of 7; it was not significantly correlated with parent weight (Fig 3A, *N* = 25, *P* = 0.581). However, brood weight (combined weight of all juveniles in a brood) was significantly related to parent weight (Fig 3B, *r*_*s*_ = 0.68, *P* < 0.001). The mean weight of all juveniles in a given brood varied from 5 to 275 mg, and was also significantly related to parent weight (Fig 3C, *r*_*s*_ = 0.65, *P* < 0.001). However, it was not significantly different among sampling months (Jan, Apr, May, Jun; *H* = 4.63, *P* = 0.201). The overall coefficient of variation (CV) of mean weight of all juveniles (*N* = 179) was 110.1%. Within-brood CV varied from 3.6% to 142.1% across 19 adults that brooded ≥ 2 juveniles, with a mean of 75.0%; it was not significantly correlated with parent weight (Fig 3D, *N* = 19, *P* = 0.281) or clutch size (*P* = 0.900) and was not significantly affected by sampling month (*F*_2,16_ = 0.37, *P* = 0.699). The mean among-brood CV of weight of incubated juveniles in the field was 79.4%.

### Offspring phenotype at release

Among 16 adult sea anemones (2.7–24.1 g) reared under laboratory conditions for long-term monitoring in two experimental periods (June 2009-March 2010, and April 2010-April 2011), 11 (68.8%) released juveniles naturally (premature propagules mentioned earlier were excluded from this analysis). Three sea anemones released a total of 15 juveniles in August and September 2009 and eight released a total of 43 juveniles from August-October 2010 (Table A in S1 File). Weights of these naturally-released juveniles were 2–311 mg, with a mean of 76.2 mg. Among nine parents that released ≥ 2 juveniles, the mean weight of juveniles released varied from 7 to 271 mg, and was not significantly related to either adult weight (*P* = 0.516), clutch size (*P* = 0.813), month (Jul, Aug, Sep; *F*_2,6_ = 0.16, *P* = 0.856) or year (*t* = 0.56, *P* = 0.596). The overall CV of mean weight of all juveniles (*N* = 57) was 84.3%. Within-brood CV of mean juvenile weight was 7.2–87.9% (mean of 40.5%). It was not related to parent weight (*N* = 9, *P* = 0.488) or clutch size (*P* = 0.498), and did not vary significantly across months (*F*_2,6_ = 0.371, *P* = 0.704) or years (*t* = 0.13, *P* = 0.900). The among-brood CV of weight of naturally-released juveniles in the laboratory was 100.7%.

### Pre-release and at-release comparisons of offspring phenotype

At the end of the monitoring periods, in March 2010 and April 2011, 12 out of 16 adults (75%) were still brooding juveniles (> 6 months after the last natural release). There were 1–16 juveniles per brood, for an overall total of 98 (Table A in S1 File). Their weight was 1–296 mg, with a mean of 34 mg. A single infertile adult was identified; it did not release juveniles during the monitoring period and was not brooding at the end of the study. For the 10 parents that brooded ≥ 2 juveniles, mean juvenile weight was 11–118 mg, with an overall mean of 44 mg; there was no evidence that it varied significantly based on adult weight (*P* = 0.160), clutch size (*P* = 0.089), or year (*U* = 3.00, *P* = 0.060). The overall CV of mean weight of all juveniles (*N* = 96) was 131.7%. The within-brood CV of juvenile weight was 21.2–144.8% (mean of 83.6%); there was no indication that it was significantly related to adult weight (*P* = 0.275), clutch size (*P* = 0.098) or year (*t* = -2.22, *P* = 0.058). In addition, within-brood CV of mean weight of brooded juveniles from parents maintained under captive conditions for about one year was not significantly different to that of brooded juveniles from parents examined immediately after collection from the field (*t* = 0.61, *df* = 27, *P* = 0.546). The among-brood CV of weight of incubated juveniles in the laboratory was 84.6%.

At the population level, the overall CV of mean weight of juveniles (whether incubated or released) was consistently high (>67%). During incubation, the CV within-brood was either similar to or higher than the CV among-brood (both in the field and in the laboratory); whereas the opposite occurred at release (in the laboratory), i.e. the CV within broods was consistently higher than the CV among broods. Overall, the CV of mean juvenile weight within broods was twofold higher before release than at release; inversely, the CV among broods was twofold higher at release than during incubation.

### Intra-brood feeding

Four adults (out of six) were brooding two or three juveniles (total of nine) at the end of this study. The proportion of juveniles that fed on food ingested by the adult (ratio of juveniles with traces of feeding to the total number of brooded juveniles) was 50–100%. The mean weight of juveniles with traces of feeding (134 ± 59 mg, ± SD, *N* = 6) was greater than that of juveniles without any trace of feeding (58 ± 39 mg; *N* = 3), noting that the weight of food traces was negligible relative to juvenile weight. However, this difference was not statistically significant (*t* = 2.00, *P* = 0.086).

### Lipid composition and fatty acid profiles

Adult tissues (body wall and oogenic mesenteries) and juveniles (large and small) were composed mainly of phospholipids (PL), sterols (ST), acetone mobile polar lipids (AMPL), triacylglycerols (TG), free fatty acids (FFA), hydrocarbons (HC), ethyl ketones (EK) and methyl esters (ME) (Table B in S1 File). Total lipid content (mean ± SE) accounted for 2.0 ± 0.2% of wet weight in adult body wall, 4.0 ± 0.2% in oogenic mesenteries, 3.3 ± 0.4% in large juveniles, and 5.0 ± 0.6% in small juveniles. Because lipids and fatty acids have not previously been studied in the genus *Aulactinia*, we provide a more complete outline and discussion in the Supplementary Text in S1 File. Here we focus on differences across sample types.

The polar lipid classes, AMPL and PL, were the most common lipids in the four types of samples, comprising 75.2 ± 2.6% in adult body wall, 60.1 ± 1.7% in oogenic mesenteries, 66.8 ± 4.0% in large juveniles and 62.7 ± 2.8% in small juveniles. The concentration of AMPL in large juveniles was not significantly different from that in the two types of adult tissue, but the concentration in small juveniles was significantly higher than that in adult body wall (Table B in S1 File). Proportions of AMPL did not vary significantly among the four types of samples. The concentrations of PL in large juveniles and small juveniles were not significantly different from those in oogenic mesenteries, but were significantly higher than in adult body wall. PL proportion in large juveniles was not significantly different from that in the two types of adult tissues; whereas PL proportion in small juveniles was significantly higher than in adult body wall (Table B in S1 File).

Among some 50 fatty acids (FA) identified in the samples, there were 24 major ones (> 1% in at least one type of sample; Table C in S1 File), that accounted for > 90% of total FA in adult body wall, oogenic mesenteries, and juveniles. The proportion of polyunsaturated fatty acids (ΣPUFA), the most common FA group, was similar in all sample types (Table C in S1 File). Proportions of most major PUFAs were similar in large and small juveniles, except 20:2a and 20:5n-3 (EPA). EPA was the major PUFA in all samples, and its level in large juveniles was similar to that in the two types of adult tissue, but was significantly higher than in small juveniles. Besides EPA, the PUFAs that represented > 5% were 22:4n-6, 22:5n-3, and the essential fatty acids 20:4n-6 (ARA) and 22:6n-3 (DHA).

**Fig 4:**
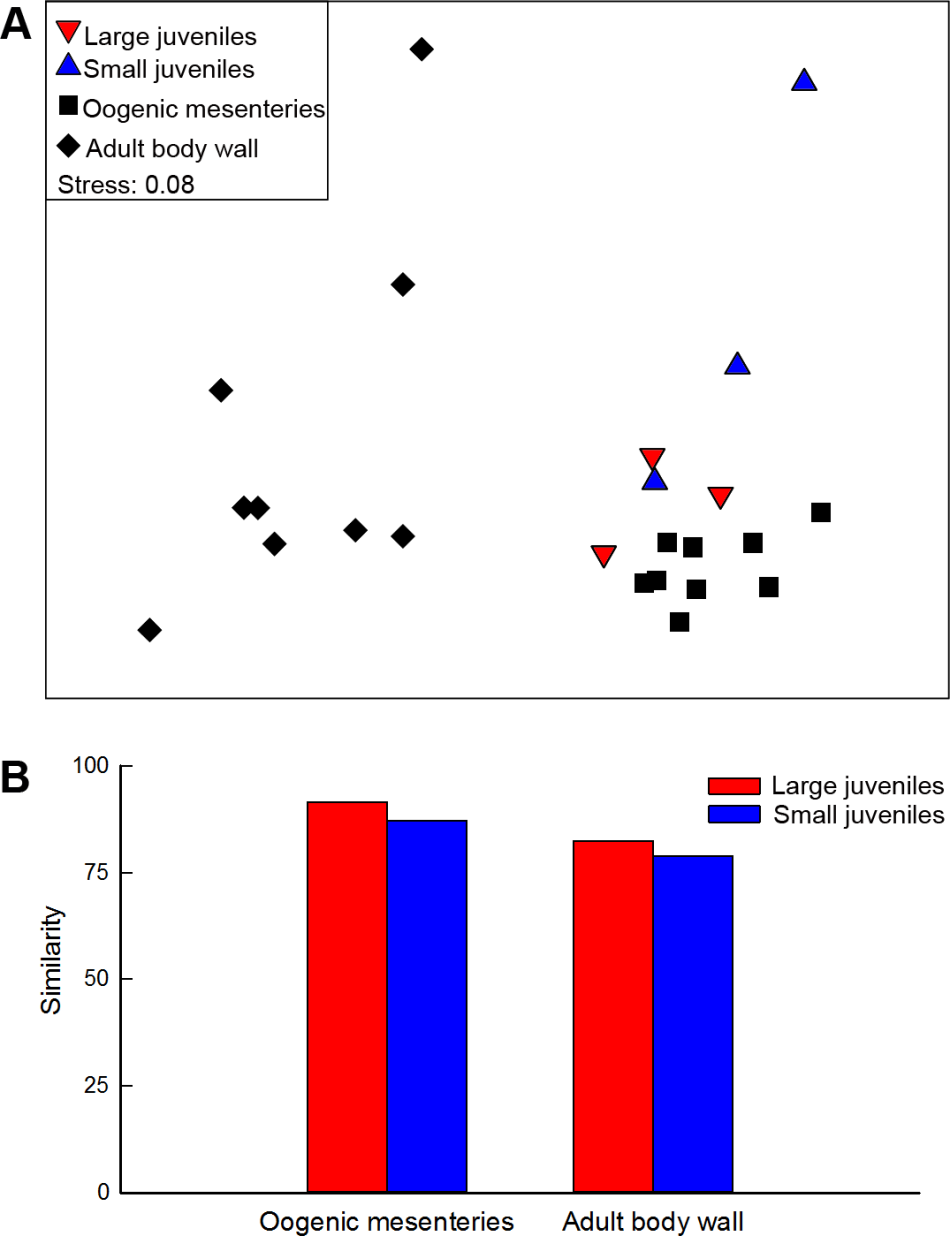
Fatty acid analysis of adult and juvenile tissues in *Aulactinia stella*. (A) Multidimensional scaling (MDS) 2-D plot of Bray-Curtis similarity index of major fatty acids from large and small juveniles (wet weight indicated), oogenic mesenteries and adult body wall. (B) Bray-Curtis similarity index between large and small juveniles with adult tissue (oogenic mesenteries and adult body wall).

MDS showed FA distributions in large and small juveniles were more similar to oogenic mesenteries than adult body wall (Fig 4A). ANOSIM revealed that fatty acid proportions were significantly different among juveniles and adult tissue, but not between large and small juveniles (*P* = 0.10). Although fatty acids were not significantly different in large and small juveniles, R_ANOSIM_ revealed that large juveniles were more similar to adult tissue (*vs* oogenic mesenteries, *R* = 0.679; *vs* adult body wall, *R* = 0.635) than small juveniles (*vs* oogenic mesenteries, *R* = 0.744; *vs* adult body wall, *R* = 0.726). In addition, SIMPER analysis showed that similarity between large juveniles and adult tissue was greater than similarity between small juveniles and adult tissue (Fig 4B), and that essential EPA and DHA contributed to > 5% of the dissimilarity in all pairwise comparisons among different types of samples (Table D in S1 File).

## Discussion

While live-bearing is ubiquitous among animals, it is chiefly studied in vertebrate models and still poorly understood in basal metazoans [4]. The sea anemone *A*. *stella* was found to exhibit superfetation and prolonged brooding of small clutches for an indeterminate period at the culmination of which fully-formed benthic juveniles are released. The size of these juveniles varied markedly, both throughout incubation and at release, without any clear relation to parent size, clutch size, environment or month/year. Results from feeding trials and lipid and fatty acid analyses suggest shifts in offspring nutrition modes, from vitellogenic provisioning (lecithotrophy) to extra-vitellogenic nourishment (matrotrophy) to prenatal feeding facilitated by the parent (nursing), as juveniles develop functional feeding organs. In turn, parent-offspring conflicts and sibling rivalries may emerge during parental care, thereby shaping the marked offspring phenotype variations evidenced in *A*. *stella*. The novel arena presented here in a basal metazoan will be useful in exploring evolutionary concepts (e.g. viviparity-driven conflict, evolution of matrotrophy) through comparisons with analogous vertebrate systems such as placental fish [25–27].

### Asynchronous offspring development

Significant offspring size variations, typically >40% and up to 132% in the overall population, were documented in *A*. *stella*. Using Jacobs and Podolsky’s [28] conversion rate for CV measured in length *vs* volume (= weight), we find that the CV of mean juvenile size in *A*. *stella* is generally higher than in ~100 species of marine invertebrates reviewed by Marshall and Keough [29]. Interestingly, the species with a comparably high CV is a live-bearing holothuroid echinoderm, emphasizing the unique nature of this reproductive strategy, which is taxonomically widespread [4]. Further research will be required to determine if and why certain forms of incubation drive marked offspring size variations. Exploring the case of *A*. *stella* already provides some insight.

Attempts have been made to relate offspring size plasticity to bet hedging, a concept that has received much attention (mainly in Chordata and Arthropoda) but remains hard to assess [30]. *A*. *stella* is a long-lived sessile species that produces fully-developed philopatric juveniles. Adults may thus be able to anticipate some properties of the environment that will be faced by juveniles. The among-brood variance in *A*. *stella* was higher than the mean within-brood variance for newly-released juveniles, as generally predicted under such conditions. However, an inverse trend was observed for competent/viable juveniles sampled in the gastrovascular cavity of sea anemones from the field, irrespective of the time of collection (i.e. CV_within_ ≥ CV_among_ at any time during incubation). Other maternal effects commonly identified as determinants of offspring size plasticity include maternal size and experience [29, 31]. Here, no evidence was found of a relationship between within-brood CV of juvenile size and either parent size or clutch size, indicating that adult phenotype is probably not the primary driver of offspring size variation in *A*. *stella*. Variance appeared to be similar whether measured in the broods of adults that were freshly collected from the field in different months or in the broods of adults maintained for ~1 year in (comparatively benign) laboratory conditions. Thus, there was no clear evidence of environmental effects either.

Factors beyond initial parental investment emerge as key mediators of offspring size plasticity in *A*. *stella* (discussed in later sections). The relative contributions of pre- and post-fertilization provisioning have previously been shown to explain inter-population offspring size variations in viviparous fish [32]. Moreover, the predominant influence of post-hatching parental care on offspring growth, which can mask the weaker effect of initial provisioning (egg size) has been experimentally demonstrated in beetle larvae that feed autonomously but can beg parents for food [33].

Another form of post-fertilization offspring size variation driven by sibling interactions has been reported in the sympatric sea anemone *Urticina felina*. Brooded embryos of *U*. *felina* can fuse with their siblings to form larger mega-larvae, which exhibit better survival to settlement [34–36]. This mechanism is unlikely to occur in *A*. *stella* given its much lower fecundity and the fact that the major differences in offspring size mirrored developmental changes. Taken together, these findings suggest that various forms of plasticity in offspring phenotype can be expected to arise in the many taxa that incubate their progeny.

### Gestational mode and shifting nourishment sources

Brooded juveniles and oogenic mesenteries were detected at all sampling dates in *A*. *stella*, including six months after the main release season, indicating (1) a prolonged brooding period, (2) overlap between brooding and oogenesis, and (3) brooding of more than one cohort of juveniles per year, with apparent generation overlap (superfetation). Lipid and fatty acid analyses support the assumption that juveniles of *A*. *stella* undergo a dietary shift during parental care. EPA and DHA, which are important for early development in marine invertebrates [21], were the most important discriminating fatty acids among samples. The proportion of EPA was significantly higher in large juveniles and oogenic mesenteries than in small juveniles, which may reflect catabolism or conversion of EPA during early development or early growth and conservation of EPA during later growth. Conservation of EPA during metabolism, indicated by high EPA content, has also been suggested in the sympatric bivalve *Yoldia hyperborea* [37]. Furthermore, similarity analyses on the major fatty acids revealed that large juveniles clustered closer to the adult tissues than to smaller juveniles, suggesting that the larger juveniles feed in a similar manner as adults, whereas younger siblings presumably assimilate dissolved nutrients.

In species that incubate their progeny, the physical and nutritional relationships between parent and offspring can be defined in various ways, giving rise to a broad terminology [1, 4, 14, 38, 39]. In the present study, small juveniles < 10 mg were not well developed (i.e. had less functional tentacles and digestive system) and had lipid signatures consistent with a dependence on pre-zygotic provisioning (lecithotrophy) and/or dissolved nutrients provided by the parent (matrotrophy). Matrotrophic brooding and viviparity have been reported in most animal phyla, including both vertebrate and invertebrate taxa [4]. Matrotrophy is believed to have evolved from lecithotrophy repeatedly [14, 39, 40], and is suggested to have done so in response to high food availability exceeding energy requirements for maintaining fairly large broods in fish [41] and reptiles [42]. The type of matrotrophy displayed by *A*. *stella* is most likely histotrophy, but may also be a form of placentotrophy if the embryos or offspring are linked/appressed to the mesenteries at any point during the incubation [4]. Histological or ultrastructural analysis will be required to confirm this.

The MDS plot showed that the largest of the ‘small’ juveniles (77 mg, able to actively feed) in *A*. *stella* was more similar to large juveniles and adult tissue than to its smaller siblings weighing 8 and 10 mg, consistent with a final shift toward semi-independent feeding. Feeding on particulate matter in the gastrovascular cavity is reminiscent of juvenile nourishment in the mantle cavity of bivalves [43, 44]. As they grow and develop prehensile and digestive organs, the incubated juveniles of *A*. *stella* apparently start to feed more readily on the food captured by the parent, evoking a form of nursing akin to the postnatal parental care seen in many vertebrates.

### Benefits and costs of brooding

While the benefits of brooding are often considered to offset low fecundity, it has recently been proposed that low fecundity is directly selected for, following the tenets of the size-number trade-off, and that egg retention is an indirect consequence of this selection [3]. In turn, offspring retention and parental care tend to increase potential for parent-offspring conflicts and sibling rivalries [3, 7, 45, 46]. In addition, matrotrophy has been suggested to prevent adaptive offspring size-number adjustments in environments characterized by fluctuations in resource availability [26]. Based on the latter, the absence of environmental control over offspring size variation in *A*. *stella* provides further support for the occurrence of matrotrophy.

The ‘safe harbour’ hypothesis initially elaborated by Shine [47, 48] makes the assumption that parental care enhances the survivorship of brooded life stages; this may occur through parental food provisioning and protection against predators [5]. In *A*. *stella*, brooding may protect soft-bodied offspring by (1) offering a barrier against environmental stressors, (2) allowing increment in size during parental care, as suggested by size-dependent survival of juveniles against specialized predators [36], and (3) timing release to decrease predation pressure. The release of offspring in *A*. *stella* occurs chiefly in August-September, at a time when their specialized predator, the nudibranch *Aeolidia papillosa*, is scarce or absent [49]. Alternatively, predator swamping has been proposed to explain the coincidence of offspring release by brooders with mass broadcast-spawning events [50].

Important costs to the mother have been documented in brooding marine invertebrates [51], which affect investment in gametes and determine the trade-off between the cost of brooding and capacity to produce eggs [52]. In *A*. *stella*, the cost of brooding is compounded by the fact that juveniles are able to consume part of the food that brooding adults obtain (i.e. parent-offspring competition), which could partly explain the generally small clutch sizes. Experimental studies on food availability and clutch size variations are needed to confirm this quantitatively. Interestingly, brooding adults of *A*. *stella* were able to release viable offspring at any time when being teased or when their body wall was damaged, indicating that the length of the brooding period is not fixed, despite the occurrence of a peak release season. This trait likely minimizes the risk of instantaneous brood mortality through parent mortality/predation associated with viviparity [53].

Overall, *A*. *stella* emerges as a singular example of reproductive strategy. Its intermediate positioning in terms of offspring nourishment (lecithotrophy, matrotrophy) and developmental state at hatching/release (precocial, altricial) likely reflects its basal position in the animal tree of life. How the shifting dietary relationship evidenced here between parent and offspring compares with known vertebrate and invertebrate matrotrophs [4, 12] calls for further investigation. The intensification of matrotrophy in live-bearing fish was recently postulated to drive shifts in sexual selection towards decreased mate choice and increased superfetation [25]. Like poeciliid fish, actiniid sea anemones display marked variations in the degree and timing of maternal provisioning, which could similarly be contrasted. It would also be valuable to explore whether within-brood kinship affects the level of competition and consequent offspring size variance, as noted in placental fish [27]. Reports of clonal reproduction and swapping of juveniles among mothers in *A*. *stella* [16] add a fascinating dimension to the prospect. Future work on this species and similar understudied models from basal clades could be instrumental in broadening our understanding of the effects of density and age-dependent factors on family conflicts, clutch size and offspring phenotypic plasticity.

## Supporting Information

S1 File Supplementary tables and text. **Table A,** Number and wet weight (Ww; mean ± SD) of naturally-released and brooded juveniles in adult *Aulactinia stella* of various sizes. **Table B,** Mean concentration and proportion of lipids in adult basal disk, oogenic mesenteries and large and small brooded juveniles of the sea anemone *Aulactinia stella*. Values (mean ± SE) in the same row with different superscript letters are significantly different (one-way ANOVA, p < 0.05). **Table C,** Major fatty acids (> 1% of total fatty acids) in adult basal disk, oogenic mesenteries, large and small brooded juveniles of the sea anemone *Aulactinia stella*. Values (mean ± SE) in the same row with different superscript letters are significantly different (one-way ANOVA, p < 0.05). **Table D,** Discriminating fatty acids (>5% weight) of the dissimilarity in different samples of *Aulactinia stella*. **Supplementary Text,** Discussion on lipids and fatty acids in *Aulactinia stella*.
(PDF)
