# Supplementary material for: Remarkable Shifts in Offspring Provisioning during Gestation in a Live-Bearing Cnidarian
Source: PLoS One. 2016 Apr 22;11(4):e0154051. doi: 10.1371/journal.pone.0154051 (PMC4841577; doi:10.1371/journal.pone.0154051)
Supplement: S1 File — Table A, Number and wet weight (Ww; mean ± SD) of naturally-released and brooded juveniles in adult Aulactinia stella of various sizes. Table B, Mean concentration and proportion of lipids in adult basal disk, oogenic mesenteries and large and small brooded juveniles of the sea anemone Aulactinia stella. Values (mean ± SE) in the same row with different superscript letters are significantly different (one-way ANOVA, p < 0.05). Table C, Major fatty acids (> 1% of total fatty acids) in adult basal disk, oogenic mesenteries, large and small brooded juveniles of the sea anemone Aulactinia stella. Values (mean ± SE) in the same row with different superscript letters are significantly different (one-way ANOVA, p < 0.05). Table D, Discriminating fatty acids (>5% weight) of the dissimilarity in different samples of Aulactinia stella. Supplementary Text, Discussion on lipids and fatty acids in Aulactinia stella. (PDF) [file pone.0154051.s001.pdf]

Supporting Information

Supplementary Tables and Text

Remarkable Shifts in Offspring Provisioning during Gestation in a Live-bearing Cnidarian

Annie Mercier[1*], Zhao Sun[1], Christopher C. Parrish[1], Jean-François Hamel[2]

[1]*Department of Ocean Sciences, Memorial University,*
St. John's, Newfoundland and Labrador, Canada

[2] *Society for the Exploration and Valuing of the Environment (SEVE),*
Portugal Cove-St. Philips, Newfoundland and Labrador, Canada

* *Corresponding author*
E-mail: amercier@mun.ca

Table A. Number and wet weight (Ww; mean ± SD) of naturally-released and brooded juveniles in adult *Aulactinia stella* of various sizes

		Naturally-released juveniles		Brooded juveniles	
Ind[a]	Adult Ww (g)	Number	Ww (mg)	Number	Ww (mg)
I - 1[b]	13.7	3	21.0 ± 11.0	2	118.1 ± 61.0
I - 2	8.1	2	271.0 ± 32.5	0	-
I - 3	9.5	10	38.1 ± 24.1	1	82.2
I - 4	13.3	0	-	3	20.2 ± 5.9
I - 5	15.4	0	-	7	97.9 ± 98.6
I - 6	12.2	0	-	10	65.3 ± 13.8
I - 7	9.9	0	-	1	31.0
I - 8	8.5	0	-	4	40.5 ± 39.5
II - 2[b]	24.1	2	52.0 ± 5.6	14	32.6 ± 42.3
II - 3[b]	2.7	9[c]	40.1 ± 35.3	11	11.3 ± 8.6
II - 4[b]	7.4	4[d]	162.8 ± 11.8	15	20.7 ± 21.0
II - 5[b]	15.2	5	115.8 ± 9.5	14	22.2 ± 32.2
II - 8[b]	12.8	3	7.0 ± 5.0	16	13.2 ± 11.1
II - 6	13.0	1	311.0	0	-
II - 1	10.4	19	74.1 ± 37.9	0	-
II - 7	5.8	0	-	0	-
III-1	3.7	-	-	1	118.0
III-2	4.2	-	-	1	2.0
III-3	15.4	-	-	0	-
III-4	14.1	-	-	1	162.0
III-5	7.5	-	-	1	275.0
III-6	7.2	-	-	0	-
III-7	6.7	-	-	0	-
III-8	1.4	-	-	0	-
III-9	5.8	-	-	5	26.2 ± 37.5
III-10	3.2	-	-	0	-
III-11	2.1	-	-	0	-
III-12	2.3	-	-	15	8.3 ± 5.5
III-13	56.0	-	-	2	165.5 ± 113.8
III-14	10.5	-	-	0	-
III-15	22.3	-	-	2	70.5 ± 81.3
III-16	10.2	-	-	3	95.0 ± 3.5
III-17	23.4	-	-	0	-
III-18	16.0	-	-	6	83.7 ± 29.7
III-19	13.5	-	-	0	-
III-20	9.0	-	-	0	-
III-21	19.4	-	-	2	100.5 ± 34.6
III-22	13.3	-	-	5	133.6 ± 118.3
III-23	16.1	-	-	8	127.3 ± 90.7
III-24	1.8	-	-	3	5.2 ± 3.7
III-25	1.2	-	-	1	0.5
III-26	7.5	-	-	6	139.7 ± 136.3
III-27	20.1	-	-	7	166.4 ± 74.1

III-28	3.9	-	-	0	-
III-29	1.1	-	-	0	-
III-30	24.2	-	-	0	-
III-31	20.7	-	-	0	-
III-32	9.3	-	-	8	53.4 ± 39.6
III-33	4.2	-	-	4	18.0 ± 24.4
III-34	3.1	-	-	0	-
III-35	32.7	-	-	57	53.0 ± 32.7
III-36	7.3	-	-	11	17.6 ± 16.4
III-37	4.6	-	-	6	18.2 ± 12.4
III-38	4.2	-	-	5	7.6 ± 4.5
III-39	18.8	-	-	1	39.0
III-40	9.2	-	-	18	40.8 ± 37.8

a: Prefix I- identifies individuals that were monitored from June 2009 to March 2010, II- individuals that were monitored from April 2010 to April 2011, and III- individuals that were freshly collected from the field in March-June 2010 and January 2011.

b: Individuals shown in Fig. 4.

c: Not including 14 tiny propagules released in mucus bundles in July and October 2010.

d: Not including 11 tiny propagules released in August 2010.

Table B. Mean concentration and proportion of lipids in adult basal disk, oogenic mesenteries and large and small brooded juveniles of the sea anemone *Aulactinia stella*. Values (mean ± SE) in the same row with different superscript letters are significantly different (one-way ANOVA, $p < 0.05$)

Lipids	Adult basal disk (n = 11)		Oogenic mesenteries (n = 9)		Large juveniles (n = 6)		Small juveniles (n = 6)	
	Concentration (µg mg^{-1})	Proportion (%)	Concentration (µg mg^{-1})	Proportion (%)	Concentration (µg mg^{-1})	Proportion (%)	Concentration (µg mg^{-1})	Proportion (%)
Hydrocarbons	0.28 ± 0.07 ^{a}	1.47 ± 0.39 ^{AB}	0.30 ± 0.07 ^{a}	0.73 ± 0.13 ^{B}	0.49 ± 0.08 ^{ab}	1.52 ± 0.25 ^{AC}	6.30 ± 3.22 ^{b}	9.98 ± 4.28 ^{C}
Methyl Esters	0.21 ± 0.04 ^{a}	1.03 ± 0.16 ^{A}	0.96 ± 0.11 ^{b}	2.41 ± 0.24 ^{AB}	1.49 ± 0.63 ^{b}	4.24 ± 1.25 ^{B}	1.12 ± 0.42 ^{b}	2.44 ± 0.90 ^{AB}
Ethyl Ketones	0.21 ± 0.09 ^{a}	1.10 ± 0.47 ^{A}	0.86 ± 0.17 ^{ab}	2.10 ± 0.32 ^{A}	1.26 ± 0.72 ^{b}	3.43 ± 1.45 ^{A}	1.64 ± 0.32 ^{b}	3.17 ± 0.49 ^{A}
Triacylglycerols	0.38 ± 0.07 ^{a}	1.98 ± 0.47 ^{A}	8.11 ± 0.72 ^{b}	20.20 ± 1.34 ^{B}	3.39 ± 0.89 ^{c}	9.85 ± 1.61 ^{C}	3.20 ± 0.97 ^{c}	6.92 ± 2.00 ^{C}
Free Fatty Acids	0.38 ± 0.10 ^{a}	1.89 ± 0.43 ^{A}	0.64 ± 0.31 ^{a}	1.56 ± 0.58 ^{A}	0.16 ± 0.11 ^{a}	0.44 ± 0.28 ^{A}	2.40 ± 1.08 ^{a}	4.50 ± 2.07 ^{A}
Sterols	3.20 ± 0.31^{a}	16.25 ± 1.49 ^{A}	4.67 ± 0.73 ^{a}	11.38 ± 1.17 ^{B}	3.55 ± 0.27 ^{a}	11.36 ± 0.94 ^{B}	3.25 ± 0.61 ^{a}	7.94 ± 2.64 ^{B}
Acetone Mobile Polar Lipids	0.87 ± 0.17 ^{a}	4.93 ± 1.20 ^{A}	1.41 ± 0.21 ^{ab}	3.49 ± 0.40 ^{A}	1.32 ± 0.33 ^{ab}	4.44 ± 1.23 ^{A}	3.47 ± 0.70 ^{b}	6.73 ± 0.82 ^{A}
Phospholipids	14.36 ± 1.38 ^{a}	70.25 ± 2.86 ^{A}	22.54 ± 0.95 ^{bc}	56.58 ± 1.75 ^{B}	20.35 ± 2.85 ^{c}	62.4 ± 3.29 ^{AB}	27.15 ± 2.91 ^{b}	55.95 ± 2.98 ^{B}
Total	20.19 ± 1.48 ^{a}	--	39.57 ± 1.93 ^{b}	--	32.58 ± 4.33 ^{b}	--	49.75 ± 6.79 ^{c}	--

Table C. Major fatty acids (> 1% of total fatty acids) in adult basal disk, oogenic mesenteries, large and small brooded juveniles of the sea anemone *Aulactinia stella*. Values (mean ± SE) in the same row with different superscript letters are significantly different (one-way ANOVA, $p < 0.05$)

% Fatty acids*	Adult basal disk (n = 9)	Oogenic mesenteries (n = 9)	Large juveniles (n = 3)	Small juveniles (n = 3)
ι17:0	1.59 ± 0.12^{a}	0.77 ± 0.10^{b}	1.05 ± 0.07^{b}	1.04 ± 0.08^{b}
16:0	5.06 ± 0.61^{a}	6.82 ± 0.14^{b}	5.50 ± 0.19^{ab}	6.29 ± 1.76^{ab}
18:0	5.6 ± 0.39^{a}	5.48 ± 0.12^{a}	1.57 ± 0.20^{a}	6.43 ± 0.78^{a}
ΣSFA	15.26 ± 1.20^{a}	15.38 ± 0.27^{a}	14.03 ± 0.10^{a}	16.07 ± 3.39^{a}
15:1	2.71 ± 0.15^{a}	1.05 ± 0.14^{b}	1.43 ± 0.45^{b}	1.49 ± 0.08^{b}
16:1n-7	1.62 ± 0.17^{a}	2.39 ± 0.06^{b}	1.87 ± 0.13^{a}	2.69 ± 0.10^{b}
18:1n-9	1.29 ± 0.40^{a}	2.38 ± 0.29^{b}	2.06 ± 0.18^{ab}	3.25 ± 0.25^{b}
18:1n-7	1.46 ± 0.24^{a}	2.89 ± 0.05^{b}	2.68 ± 0.39^{b}	2.50 ± 0.14^{b}
18:1n-5? **	6.15 ± 0.28^{a}	5.36 ± 0.11^{a}	5.81 ± 0.88^{a}	5.00 ± 0.11^{a}
20:1n-11?	0.66 ± 0.13^{a}	1.06 ± 0.06^{b}	1.00 ± 0.20^{ab}	1.76 ± 0.06^{c}
20:1n-9	0.62 ± 0.09^{a}	1.73 ± 0.06^{b}	1.69 ± 0.27^{ab}	2.72 ± 0.44^{b}
20:1n-7?	1.74 ± 0.13^{a}	2.59 ± 0.06^{b}	2.58 ± 0.24^{b}	2.29 ± 0.10^{b}
22:1n-9	6.94 ± 0.33^{a}	3.49 ± 0.10^{b}	4.07 ± 0.15^{bc}	4.53 ± 0.44^{c}
22:1n-7	1.93 ± 0.16^{a}	0.45 ± 0.12^{b}	0.71 ± 0.35^{bc}	1.28 ± 0.14^{c}
ΣMUFA	26.42 ± 0.92^{a}	24.92 ± 0.3^{a}	25.50 ± 0.63^{a}	30.17 ± 1.39^{b}
16:2n-4	1.59 ± 0.12^{a}	0.45 ± 0.02^{b}	0.73 ± 0.07^{b}	0.66 ± 0.04^{b}
16:3n-4?	3.68 ± 0.26^{a}	1.72 ± 0.21^{b}	2.44 ± 0.69^{b}	1.70 ± 0.48^{b}
16:4n-3?	1.13 ± 0.15^{a}	0.44 ± 0.06^{b}	0.62 ± 0.15^{b}	0.47 ± 0.05^{b}
16:4n-1	1.28 ± 0.12^{a}	0.44 ± 0.06^{b}	0.46 ± 0.12^{b}	0.75 ± 0.19^{b}
20:2a?	0.74 ± 0.10^{a}	0.91 ± 0.09^{a}	0.75 ± 0.12^{a}	1.45 ± 0.38^{b}
20:2n-6	0.47 ± 0.06^{a}	1.08 ± 0.05^{b}	0.98 ± 0.18^{b}	0.93 ± 0.07^{b}
20:4n-6 ARA	5.00 ± 0.42^{a}	3.57 ± 0.18^{b}	3.37 ± 0.34^{b}	3.52 ± 0.23^{b}
20:5n-3 EPA	24.32 ± 1.11^{ab}	27.77 ± 0.35^{b}	28.01 ± 0.41^{b}	22.37 ± 2.46^{a}
22:4n-6?	8.56 ± 0.59^{a}	6.73 ± 0.35^{b}	5.28 ± 0.59^{b}	6.04 ± 0.49^{b}
22:5n-3	6.60 ± 0.34^{a}	8.87 ± 0.19^{b}	7.27 ± 0.61^{a}	7.77 ± 0.31^{ab}
22:6n-3 DHA	1.75 ± 0.18^{a}	4.15 ± 0.40^{b}	5.25 ± 0.66^{bc}	5.74 ± 0.63^{c}
ΣPUFA	58.32 ± 2.08^{a}	59.7 ± 0.42^{a}	60.48 ± 0.64^{a}	53.76 ± 4.51^{a}
Bacterial	6.80 ± 0.31^{a}	3.66 ± 0.15^{b}	4.37 ± 0.54^{b}	4.29 ± 0.05^{b}
P/S	4.04 ± 0.35^{a}	3.89 ± 0.09^{a}	4.31 ± 0.06^{a}	3.74 ± 0.96^{a}
Σn-3	35.12 ± 1.48^{a}	43.05 ± 0.63^{b}	38.12 ± 4.19^{ab}	43.21 ± 1.47^{b}
DHA/EPA ratio	0.07 ± 0.01^{a}	0.15 ± 0.02^{b}	0.23 ± 0.01^{c}	0.21 ± 0.02^{c}

* Only major fatty acids (>1%) are reported. Minor fatty acids include: 14: 0, ι15:0, αι15:0, 15:0, ι16:0, αι16:0?, 17:0, αι17:0, 20:0, 22:0, 14:1, 16:1n-5, 16:1n-9?, 17:1,18:1n-11?, 20:1n-9, 20:1n-7?, 22:1n-11(13), 24:1, 18:2n-6, 18:2n-4, 18:3n-6, 18:3n-4, 18:3n-3, 18:4n-3, 20:2b?, 20:3n-3, 20:4n-3, 21:5n-3?

**? Identity of FA not confirmed by comparison with a standard or by mass spectrometry, but by comparison with Ackman (1986) .

Table D. Discriminating fatty acids (>5% weight) of the dissimilarity in different samples of *Aulactinia stella*

Type of samples	Fatty acids	Proportion (%)	Proportion (%)	Contribution* (%)
Small juveniles *vs* Large juveniles	20:5n3 EPA	22.37	28.01	22.97
	16:0	6.29	5.5	8.36
	18:0	6.43	4.97	7.53
	22:6n3 DHA	4.79	6.2	5.77
	18:1n5?	6.03	4.77	5.15
Small juveniles *vs* Oogenic mesenteries	20:5n3 EPA	22.37	27.77	22.89
	16:0	6.29	6.82	10.44
	18:0	6.43	5.48	7.63
	22:5n3	7.19	8.87	7.16
	22:4n6?	5.45	6.73	6.49
	22:6n3 DHA	4.79	4.15	5.31
Large juveniles *vs* Oogenic mesenteries	22:6n3 DHA	6.2	4.15	13.11
	16:0	5.5	6.82	8.11
	22:4n6?	5.86	6.73	8.02
	16:3n4?	2.09	1.72	6.95
	22:1n9	4.53	3.49	6.46
	22:5n3	7.86	8.87	6.27
	20:5n3 EPA	28.01	27.77	5.64
	22:1n7	1.31	0.45	5.29
Small juveniles *vs* Adult body wall	20:5n3 EPA	22.37	24.32	10.54
	22:4n6?	5.45	8.56	8.31
	22:6n3 DHA	4.79	1.75	7.81
	22:1n9	4.08	6.94	7.36
	16:0	6.29	5.06	6.33
	18:1n9	3.25	1.29	5.67
	20:1n9	2.71	0.62	5.38
Large juveniles *vs* Adult body wall	22:6n3 DHA	6.2	1.75	13.78
	20:5n3 EPA	28.01	24.32	11.47
	22:4n6?	5.86	8.56	8.95
	22:1n9	4.53	6.94	7.58
	20:4n6 AA	3.27	5	5.74
	16:3n4?	2.09	3.68	5.68
Oogenic mesenteries *vs* Adult body wall	20:5n3 EPA	27.77	24.32	10.09
	22:1n9	3.49	6.94	9.74
	22:6n3 DHA	4.15	1.75	6.78
	16:0	6.82	5.06	6.51
	22:5n3	8.87	6.6	6.4
	22:4n6?	6.73	8.56	6.27
	16:3n4?	1.72	3.68	5.54

* Percentage of average dissimilarity due to the fatty acid

Supplementary text

Lipids and fatty acids have not previously been studied in the genus *Aulactinia.* We therefore provide a brief discussion on this aspect here. Joseph (1979) reviewed the lipid content and composition in several species of sea anemones, and proposed that the total lipids (% of wet weight) in sea anemones were highly variable, i.e. from a low of 0.3% to a high of 5.2%. Total lipid content of *Aulactinia stella* accounted for 2.0 ± 0.2% of wet weight in adult basal disk, 3.3 ± 0.4% in large juveniles, and 5.0 ± 0.6% in small juveniles. This is in line with the observation that larger individuals have less lipids than smaller ones in the temperate sea anemone *Metridium dianthus* from the NW Atlantic (Hooper and Ackman 1971). In *A. stella* studied here, the polar lipid classes, AMPL and PL, were the most common lipids in the four types of samples, i.e. comprising more than 60% of total lipids, similar to the proportions of polar lipids in the temperate sea anemone *Metridium senile* (57.2-83.5%, Hill-Manning and Blanquet 1979).

The essential fatty acid EPA was the most abundant fatty acid (22.4-27.8% of total FAs) in all four types of samples in *A. stella,* and the values were especially high in oogenic mesenteries and large juveniles. The proportion of EPA in *A. stella* was higher than in the temperate sea anemone *Phymactis clematis* (13.9% of total FAs) collected in SW Atlantic (Mar del Plata, Argentina, Pollero 1983) and 19 sympatric species of marine invertebrates (2.3-18.4%) collected from a close sampling area, Conception Bay, Newfoundland (Parrish et al. 2009). The proportions of DHA in oogenic mesenteries and juveniles of *A. stella* (4.2-5.7% of total FAs) were within the range of the 19 sympatric species of marine invertebrates (2.31-18.4%, Parrish et al. 2009). However, the

proportion of DHA only accounted for 1.8% of total fatty acids in adult basal disk of *A. stella,* which was lower than the values in the sympatric marine invertebrates.

References

Ackman, R. G. 1986. WCOT (capillary) gas-liquid chromatography, Pages 137-206 *in* R. J. Hamilton, and J. B. Rossell, eds. Analysis of Oils and Fats London: Elsevier Applied Science.

Hill-Manning, D. N., and R. S. Blanquet. 1979. Seasonal changes in the lipids of the sea anemone, *Metridium senile* (L.). Journal of Experimental Marine Biology and Ecology 36:249-257.

Hooper, S. N., and R. G. Ackman. 1971. Trans-6-hexadecenoic acid and the corresponding alcohol in lipids in the sea anemone *Metridium dianthus*. Lipids 6:341-346.

Joseph, J. D. 1979. Lipid composition of marine and estuarine invertebrates: porifera and cnidaria. Progress in Lipid Research 18:1-30.

Parrish, C. C., D. Deibel, and R. J. Thompson. 2009. Effect of sinking spring phytoplankton blooms on lipid content and composition in suprabenthic and benthic invertebrates in a cold ocean coastal environment. Marine Ecology Progress Series 391:33-51.

Pollero, R. J. 1983. Lipid and fatty acid characterization and metabolism in the sea anemone *Phymactis clematis* (Dana). Lipids 18:12-17.
